# Supplementary material for: Using Self-Assembling ADDomer Platform to Display B and T Epitopes of Type O Foot-and-Mouth Disease Virus
Source: Viruses. 2022 Aug 18;14(8):1810. doi: 10.3390/v14081810 (PMC9416097; doi:10.3390/v14081810)
Supplement: Supplementary file 1 [file viruses-14-01810-s001.zip › viruses-1822969-supplementary.pdf]

Supplemental Figure:

A

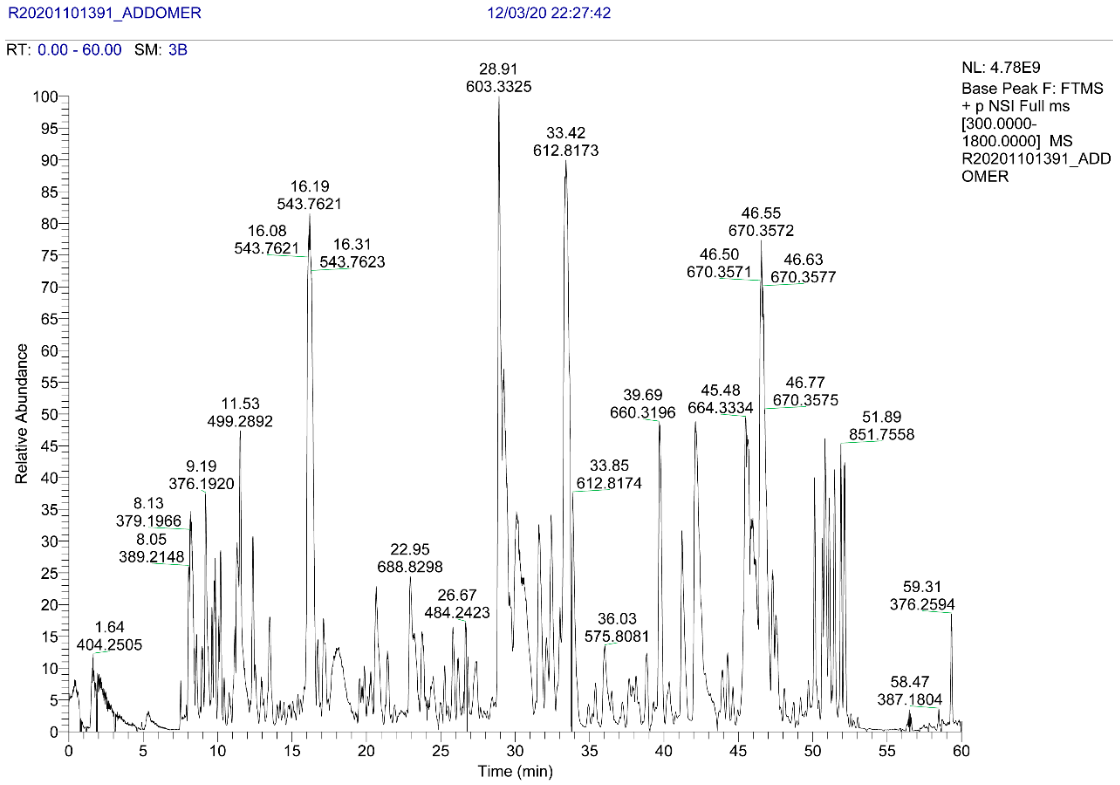

B

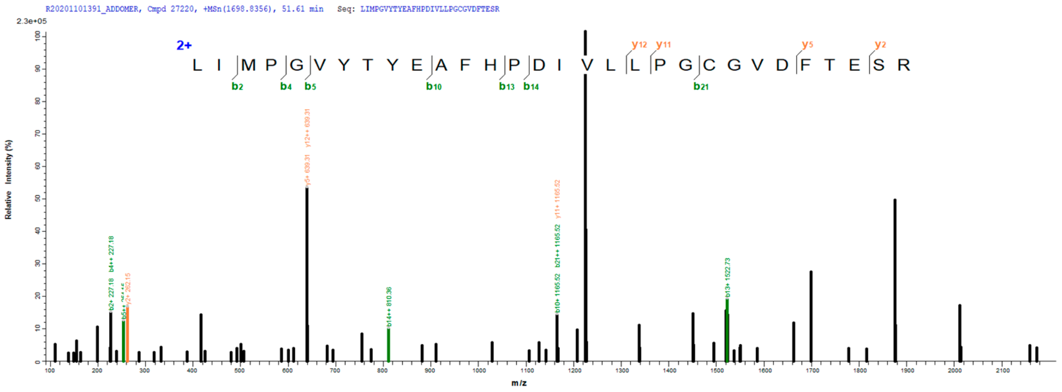

C

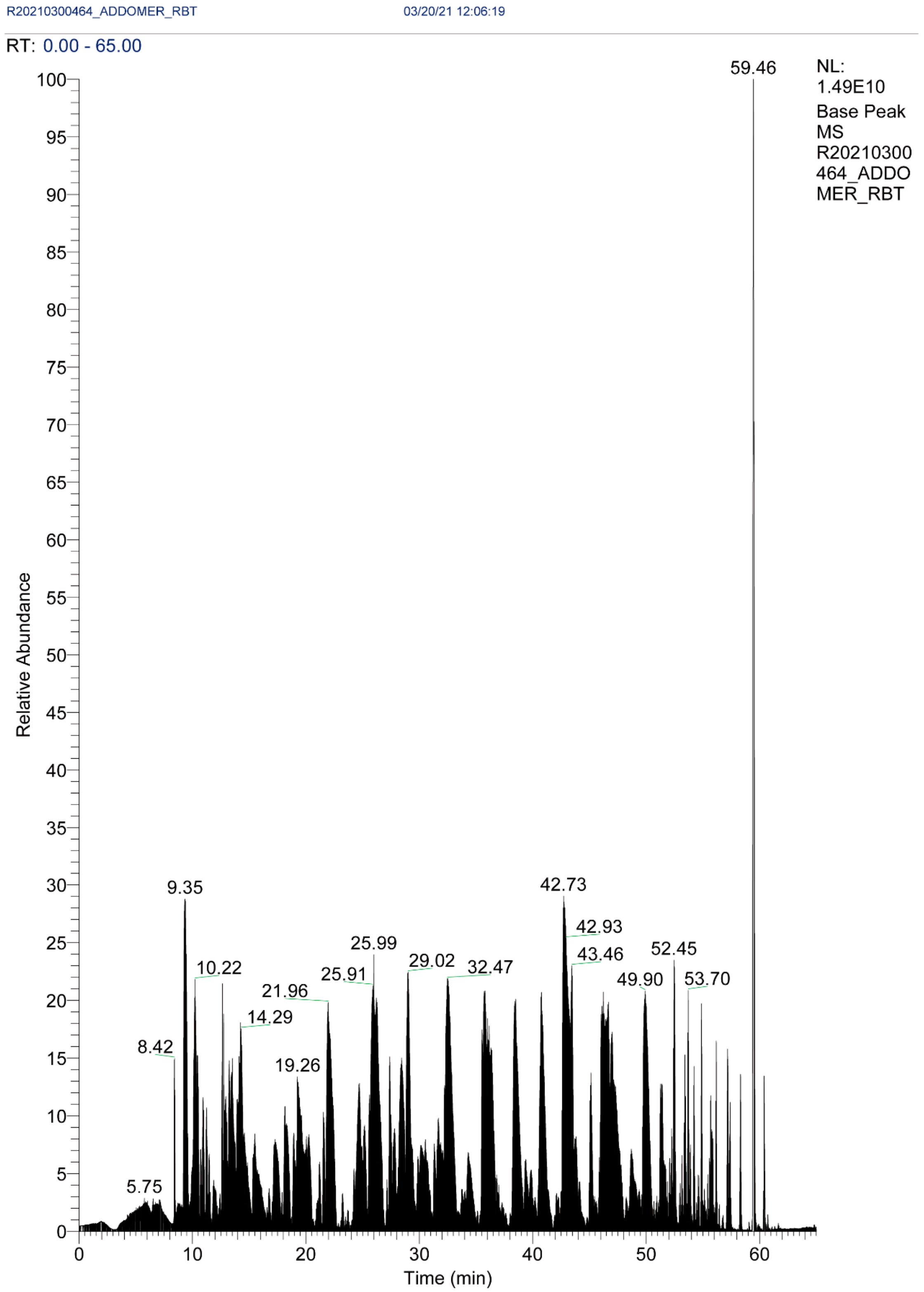
R20210300464_ADDOMER_RBT
03/20/21 12:06:19
RT: 0.00 - 65.00
NL:
1.49E10
Base Peak
MS
R20210300
464_ADDO
MER_RBT
59.46
9.35
42.73
42.93
43.46
52.45
25.99
29.02
32.47
10.22
25.91
49.90
53.70
21.96
14.29
8.42
19.26
5.75
Relative Abundance
Time (min)
0
5
10
15
20
25
30
35
40
45
50
55
60
65
70
75
80
85
90
95
100

D

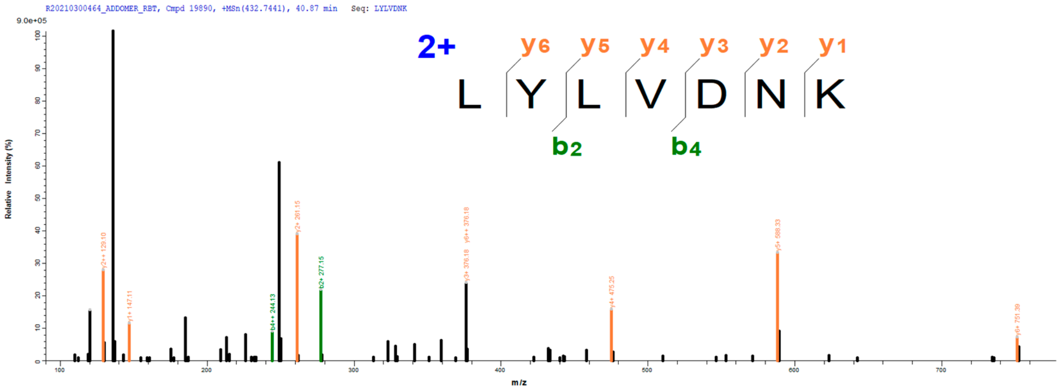

Figure:ADDomer and ADDomer-RBT mass spectrometry results. A: ADDomer protein secondary spectrum results. B: The peptide segment with the highest ADDomer score (244-275 aa) was drawn with a secondary mass spectrum. According to the peak map results, the peptide segment was successfully detected by mass spectrometry, indicating that the ADDomer protein was successfully expressed. C: ADDomer-RBT protein secondary spectrum results. D: Take the 72-80 aa of ADDomer-RBT to make a secondary peak map. According to the peak map results, it can be seen that the peptide can be detected by mass spectrometry, indicating that the ADDomer-RBT protein is successfully expressed.
